# Elemental Mapping of Human Malignant Mesothelioma Tissue Samples Using High-Speed LA–ICP–TOFMS Imaging

**DOI:** 10.1021/acs.analchem.1c04857

**Published:** 2022-01-24

**Authors:** Oana M. Voloaca, Malcolm R. Clench, Gunda Koellensperger, Laura M. Cole, Sarah L. Haywood-Small, Sarah Theiner

**Affiliations:** † Biomolecular Sciences Research Centre, Sheffield Hallam University, Howard Street, S1 1WB Sheffield, U.K.; ‡ Institute of Analytical Chemistry, Faculty of Chemistry, University of Vienna, Waehringer Straße 38, 1090 Vienna, Austria

## Abstract

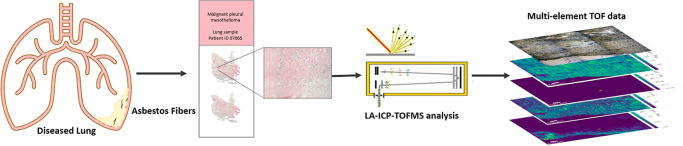

This is the first report of the use of laser ablation–inductively coupled plasma time-of-flight mass spectrometry (LA–ICP–TOFMS) to analyze human malignant pleural mesothelioma (MPM) samples at the cellular level. MPM is an aggressive, incurable cancer associated with asbestos exposure, with a long latency and poor overall survival. Following careful optimization of the laser fluence, the simultaneous ablation of soft biological tissue and hard mineral fibers was possible, allowing the spatial detection of elements such as Si, Mg, Ca, and Fe, which are also present in the glass substrate. A low-dispersion LA setup was employed, which provided the high spatial resolution necessary to identify the asbestos fibers and fiber fragments in the tissue and to characterize the metallome at the cellular level (a pixel size of 2 μm), with a high speed (at 250 Hz). The multielement LA–ICP–TOFMS imaging approach enabled (i) the detection of asbestos fibers/mineral impurities within the MPM tissue samples of patients, (ii) the visualization of the tissue structure with the endogenous elemental pattern at high spatial resolution, and (iii) obtaining insights into the metallome of MPM patients with different pathologies in a single analysis run. Asbestos and other mineral fibers were detected in the lung and pleura tissue of MPM patients, respectively, based on their multielement pattern (Si, Mg, Ca, Fe, and Sr). Interestingly, strontium was detected in asbestos fibers, suggesting a link between this potential toxic element and MPM pathogenesis. Furthermore, monitoring the metallome around the talc deposit regions (characterized by elevated levels of Al, Mg, and Si) revealed significant tissue damage and inflammation caused by talc pleurodesis. LA–ICP–TOFMS results correlated to Perls’ Prussian blue and histological staining of the corresponding serial sections. Ultimately, the ultra-high-speed and high-spatial-resolution capabilities of this novel LA–ICP–TOFMS setup may become an important clinical tool for simultaneous asbestos detection, metallome monitoring, and biomarker identification.

## Introduction

Malignant pleural mesothelioma (MPM) is an incurable malignancy associated with high symptom burden and poor prognosis. This asbestos-related cancer is characterized by a long latency period (30–60 years), developing from mesothelial cells lining the lungs and chest cavity.^1^ MPM is generally diagnosed at such a late stage that treatment options are often limited and ineffective.^2^ The overall survival varies between 5 and 13 months as a result of several factors including the absence of reliable blood and tissue MPM biomarkers,^2^ lack of effective treatment strategies,^3^ and limited detection of asbestos fibers in patient samples.^4^ Early diagnosis plays a key role in increasing the overall survival. The analysis of asbestos counts and fiber types present in tissue samples may aid early MPM diagnosis and provide insights into the pathogenesis of asbestos-related diseases. This may also be significant for differential diagnosis, risk assessment, and medicolegal compensation.^5^

Asbestos is a generic name for a range of fibrous silicate minerals, with high Mg, Ca, and Fe content, that exhibit, to a greater or lesser extent, high tensile strength and elevated resistance to heat and chemical disintegration. According to the World Health Organization classification, asbestos fibers are particles thinner than 3 μm, longer than 5 μm, and with an aspect ratio (length to width) of 3:1 or greater.^6^ Only the fibers meeting these criteria (≈5–15%) are under strict regulatory guidelines and are considered in the pathology examination despite numerous reports of short fiber toxicity.^6,7^ Due to the unique physical properties, asbestos has been extensively employed in high-resource countries including the United Kingdom, Australia, the USA, and the majority of Europe until the early 1980s. Afterward, the use of some amphibole fibers (crocidolite, actinolite, tremolite, anthophyllite, and amosite) and the serpentine fiber, chrysotile, was limited or totally banned due to their carcinogenic properties. However, asbestos continues to be a global issue, with around 125 million people worldwide being exposed to potentially hazardous mineral fibers at their workplaces, schools, or homes.^8^ Additionally, only 6 out of approximately 400 documented mineral fibers are under strict regulatory guidelines, although many of the remainder have also been linked to malignant mesothelioma.^9^ Moreover, hot spots of mesothelioma may occur concomitantly with urban development.^10^

Although the instrumentation for asbestos identification has improved significantly, the standardized sample preparation process has remained unchanged for over 3 decades.^11^ Most asbestos identification techniques, such as scanning electron microscopy and transmission electron microscopy, employ tissue digestion, meaning that the spatial information cannot be preserved. Several alternatives to monitor asbestos fibers within the biological matrix have been presented. One study by Ishida et al. observed the process of frustrated phagocytosis macrophages undergo in the presence of fluorescently labeled asbestos fibers using fluorescence microscopy.^12^ Moreover, detection based on elemental composition has also been demonstrated in several studies by Pascolo et al. using a combination of X-ray fluorescence microscopy, high-performance synchrotron X-ray fluorescence, and X-ray absorption near-edge spectroscopy analyses.^13,14^ Other techniques include energy-dispersive X-ray microanalysis and in-air micro-particle-induced X-ray emission.^4,15^ Limitations of these techniques revolve around clinical applicability, resolution, and sensitivity, as well as capabilities to acquire other elemental information *in situ*.

Laser ablation–inductively coupled plasma mass spectrometry (LA–ICPMS) is an imaging technique providing information on the multielement distribution with a high spatial resolution (down to the single-cell level) in various sample types. LA–ICPMS has been established as the workhorse for metallomics analysis of biological tissues and presents several attractive features. Recent advancements in low-dispersion LA setups have significantly improved the speed of the analysis (with a higher sample throughput) and the spatial resolution.^16,17^ We have recently demonstrated the potential of high-speed, low-dispersion LA–ICPMS setups to be employed as clinical tools for elemental mapping of asbestos and other mineral fibers within MPM cellular models.^18^ In addition, we have successfully detected and classified asbestos fibers in a three-dimensional (3D) MPM model using a fast-response LA chamber coupled to an ICP time-of-flight (TOF) MS system.^19^ However, elemental mapping of human MPM tissue samples by high-resolution LA–ICP–TOFMS has not been attempted until now. Mapping the cellular and the subcellular metallome provides insights into the fundamental biological processes and helps understand the chemical makeup during disease states. LA–ICP–TOFMS has been used as an elemental imaging tool for a variety of samples, ranging from soft biological samples^20,21^ to metamorphic and sedimentary rocks.^22^ What sets the MPM tissue samples apart is the challenge of concomitantly ablating and analyzing a soft biological matrix as well as acid-resistant mineral silicates. Human MPM tissue samples selected in this study represented different pathologies, classifications, and anatomical sites. Asbestos fibers have been detected in the tissue originating from the lungs, as human lungs having difficulties in eliminating the invasive fibers from the alveoli.^5^ Moreover, smaller mineral fibers have been previously detected in the pleura of MPM patients.^1^ Consequently, lung and pleura tissues from MPM patients were analyzed. There are no data on the presence of asbestos and other mineral fibers in the samples originating from the chest wall of MPM patients; therefore, chest wall tissue samples were also included in the sample pool for LA–ICP–TOFMS analysis. Apart from asbestos and mineral fiber detection, LA–ICP–TOFMS analysis can simultaneously provide insights into one of the hallmarks of cancer development and progression and the metal homeostasis dysregulation.

In the current study, we demonstrate the capabilities of the high-speed, low-dispersion Iridia 193 nm LA system coupled to an icpTOF 2R ICP–TOFMS instrument to spatially resolve different types of asbestos and other mineral fibers within two-dimensional (2D) cellular models of MPM and human MPM tissue sections based on their multielement pattern. Additionally, the instrumentation allows for the monitoring of the MPM metallome at the cellular level, offering further insights into a variety of perturbations in the homeostasis of metal ions that characterize neoplastic tissues. Therefore, LA–ICP–TOFMS imaging has the capability to be employed in clinical settings as a tool for mapping asbestos bodies, monitoring the MPM metallome disturbance, and detecting levels of metal-tagged MPM biomarkers, all in a single set of analysis.

## Experimental Section

### Cell Culture

Immortalized human mesothelioma cells, MSTO-211H (ATCC, UK), were cultured in a complete RPMI 1640 medium (10% v/v heat-inactivated fetal bovine serum and 1% v/v penicillin/streptomycin) at 37 °C in a humidified atmosphere with 5% CO_2_. The cells were grown and harvested following the established cell culture protocols. All cell culture materials were supplied by Gibco (Life Technologies, CA, USA). The cell line was confirmed to be negative for mycoplasma using the MycoAlert mycoplasma detection kit (LONZA, Basel, Switzerland).

### Asbestos Fibers

The Union for International Cancer Control-accredited amosite, crocidolite, and chrysotile (Health and Safety Laboratories, UK) were prepared in specially designed laminar flow hoods (Santia Asbestos Management Ltd. laboratories) in a phosphate-buffered saline (PBS) solution to a final stock concentration of 1 μg mL^–1^. Asbestos fibers pose a low risk of inhalation in an aqueous solution. The asbestos solutions were sterilized at 121 °C in an autoclave and stored at room temperature. Prior to the treatment, the solutions were passed through a 22-gauge needle five times to ensure the separation of the fibrils.

### Cell Model Development and Preparation

For the 2D MPM cell models, 1 × 10^7^ cells were harvested, centrifuged, and treated with 100 μL of the asbestos solution. Following a second centrifugation process, the models were embedded in hydroxypropyl methylcellulose/polyvinylpyrrolidone media (3:1 ratio) and flash-frozen in liquid nitrogen using specially designed plastic molds. Finally, the material was cryo-sectioned onto glass slides to produce 5 μm thick sections.

### Human Tissue Samples

The human malignant mesothelioma tissue samples were commercially obtained (AMSBIO, UK) and were purposefully selected to include a diverse pool of patients (Table S2). The fresh tumors were embedded in optimal cutting temperature media and flash-frozen prior to cryo-sectioning onto glass slides (5 μm thick sections). The tissues were supplied as serial sections and were employed for either LA–ICP–TOFMS analysis or histological staining. All data provided were shared by the supplier according to the Human Tissue Act 2004. Tissue handling and storage were carried out according to the ethical guidelines.

### Histological Staining

The human MPM tissue sections were fixed in 10% formalin for 75 min and then washed in PBS, prior to staining with either routine Mayer’s hematoxylin and eosin staining solutions (H&E) or Perls’ Prussian blue staining solutions (Advanced Testing Iron Stain, Thermo Scientific, MA, USA). For H&E staining, the slides were placed in hematoxylin for 5 min, rinsed under tap water for 5 more minutes, and then stained with eosin for 1 min. Lastly, the sections were dehydrated in three changes of absolute anhydrous alcohol for 5 min each and then cleared in three changes of a clearing agent (*i.e.*, a xylene substitute) for 5 min each. Serial sections of the same patients’ samples were used to stain for the ferric iron presence according to Perls’ Prussian blue reaction. The working iron stain solution was freshly prepared by mixing equal volumes of potassium ferrocyanide solution and hydrochloric acid solution. First, the sections were submerged in deionized water for 1 min and then stained in the working iron stain solution for 30 min at room temperature. The sections were once again washed in deionized water for 1 min before counterstaining the nuclei with a nuclear red thiazine stain solution for 30 s to achieve the desired contrast, followed by an additional deionized water wash for 30 s. Finally, the sections were dehydrated in two changes of absolute anhydrous alcohol for 1 min each and then cleared in three changes of the xylene substitute for 1 min each. Glass coverslips were mounted on the top of all the sections using Pertex mountant (CellPath Ltd., UK). The sections were imaged using an Olympus BX60 light microscope and processed and visualized using CellSens software.

### LA–ICP–TOFMS Imaging

An Iridia 193 nm LA system (Teledyne Photon Machines, Bozeman, MT, USA) was coupled to an *icp*TOF 2R ICP–TOFMS instrument (TOFWERK AG, Thun, Switzerland). The LA system was equipped with a low-dispersion ablation cell^23,24^ in a Cobalt ablation chamber and coupled with the aerosol rapid introduction system (ARIS) to the ICP–TOFMS system. An optimized He carrier gas flow of 0.60 L min^–1^ was used, and an Ar make-up gas flow of ∼0.90 L min^–1^ was introduced through the low-dispersion mixing bulb of the ARIS. Daily tuning of the instrument settings was performed using NIST SRM612 glass-certified reference material (National Institute for Standards and Technology, Gaithersburg, MD, USA) and was aimed at high sensitivity across the elemental mass range. Optimization was based on high intensities for ^24^Mg^+^, ^59^Co^+^, ^115^In^+^, and ^238^U^+^, low oxide formation based on the ^238^U^16^O^+^/^238^U^+^ ratio (<2%), and low elemental fractionation based on the ^238^U^+^/^232^Th^+^ ratio (∼1). Daily optimization included to aim at a low aerosol dispersion characterized by the pulse response duration for ^238^U^+^ based on the FW0.01 M criterion, that is, the full peak width of the ^238^U^+^ signal response obtained upon a single laser shot at 1% of the height of the maximum signal intensity.

LA sampling was performed at a repetition rate of 250 Hz using a 4 μm spot size (circular) with an interspacing of 2 μm between the lines and the individual pixels (fixed dosage mode 2), resulting in a pixel size of 2 × 2 μm. Selective ablation of the biological samples was achieved by selecting an energy density below the ablation threshold of glass and above the ablation threshold of the samples.^24^ Cell samples and tissue samples were removed quantitatively using a fluence of 1.0 J cm^–2^. Asbestos fibers were removed qualitatively with a fluence of 1.0 J cm^–2^ and quantitatively with a fluence of 3.0 J cm^–2^.

The *icp*TOF 2R ICP-TOFMS instrument has a specified mass resolution (*R* = *m*/Δ*m*) of 6000 (full width half-maximum definition) and allows the analysis of ions in the range *m/z* = 14–256. The integration and readout rate match the LA repetition rate. The instrument was equipped with a torch injector of 2.5 mm inner diameter and the nickel sample and skimmer cones with a skimmer cone insert of 2.8 mm in diameter. A radio frequency power of 1440 W, an auxiliary Ar gas flow rate of ∼0.80 L min^–1^, and a plasma Ar gas flow rate of 15 L min^–1^ were used. The instrumental parameters for the LA–ICP–TOFMS measurements are summarized in Table S1.

### Data and Image Processing

LA–ICP–TOFMS data were recorded using TOFpilot v2.10. (TOFWERK AG, Thun, Switzerland) and saved in the open-source hierarchical data format (HDF5, www.hdfgroup.org). Postacquisition data processing was performed with TofWare v3.2.2, which is a TOFWERK data analysis package and used as an add-on for IgorPro (Wavemetric Inc., Oregon, USA). The data processing included (1) drift correction of the mass peak position in the spectra over time via time-dependent mass calibration, (2) determining the peak shape, and (3) fitting and subtracting the mass spectral base line. LA–ICP–TOFMS data were further processed with HDIP (HDF-based Image Processing, Teledyne Photon Machines, Bozeman, MT, USA) software version 1.6. to generate 2D elemental images.

## Results and Discussion

### Identification of Asbestos Fibers in Cell Culture by LA–ICP–TOFMS Imaging

In previous studies, it has been shown that asbestos and other mineral fiber subsets can be differentiated within 2D and 3D mesothelioma cell culture systems based on their multielement pattern using LA–ICP–quadrupole MS and LA–ICP–TOFMS imaging.^18,19^ The major constituents of the investigated fibers are the elements Na, Mg, Al, Si, and Fe, which may also be present in the biological tissue and/or in the glass substrate. Additionally, the identification of mineral fibers within the MPM biological tissue can be challenging as it requires the ablation of the soft biological material and hard mineral material in parallel. Therefore, the selection of the laser fluence plays an important role in enabling the selective and quantitative ablation of the biological material from the glass substrate, while the laser fluence must be high enough to ablate the fiber material. In a first step, an LA–ICP–TOFMS method was evaluated in 2D cellular models using mesothelioma cells spiked with three different types of asbestos fibers. The selection of asbestos fibers included amosite, crocidolite, and chrysotile, similar to previous studies,^18,19^ as they showed different nominal compositions and degrees of carcinogenicity.^25^ The same LA parameters as those intended for the imaging experiments of the tissue samples were used to analyze the cellular models, with a pixel size of 2 μm. Different fluence levels in the range of 0.5–3.0 J cm^–2^ were evaluated, resulting in an optimized fluence of 1.0 J cm^–2^. At this fluence level, selective ablation of the cells was observed without creating imaging artifacts resulting from the coablation of the glass substrate. Subsequently, an evaluation was performed to determine whether the fluence was high enough to qualitatively ablate the asbestos fibers spiked within the cells. For the three different types of asbestos fibers, elemental levels of Na, Mg, Si, and Fe significantly above the biological background were observed. Overlays of the high phosphorus signals generated by the biological matrix and the other elements present in the mineral fibers were produced. Data suggested that crocidolite was characterized by high levels of Na, Mg, Si, and Fe, while amosite and chrysotile yielded elevated Mg, Si, and Fe signals (Figures S3 and S4). These results are in accordance with the nominal composition of the different asbestos fibers^26^ and with previous studies using the LA–ICP–sector field MS and LA–ICP–TOFMS instruments.^18,19^ Therefore, it was shown that the LA–ICP–TOFMS method and the selected fluence and laser parameters in this study are fit for the purpose of detecting asbestos fibers within human MPM tissue samples.

### High-Resolution LA–ICP–TOFMS Imaging of the Lung Tissue

High-resolution LA–ICP–TOFMS imaging was performed on MPM patient samples of different stages in the pathological presentation and tumor localization including the lung, pleura, and chest wall (Table S2). The selection of MPM tumors localized in different parts of the lung, pleura, or chest wall was essential to show not only the heterogeneity of the MPM metallome but also the asbestos fiber translocation within the respiratory system. Regions of interest (ROIs) were imaged in the lung tissue sample of patient 1 with a pixel size of 2 μm and a pixel acquisition rate of 250 Hz (enabled by the signal pulse response of the used LA setup). Again, the laser fluence was optimized to enable the selective and quantitative ablation of the biological material from the glass substrate, resulting in a fluence of 1.0 J cm^–2^. Via this approach, it was possible to image biologically essential elements present in the tissue that are also major constituents of the glass substrate, such as Na, Mg, and Ca. This approach, together with the use of an ICP–TOFMS instrument, allowed the analysis of a wide range of elements in the tissue samples including elements with key biological functions from the lower mass range, such as sodium, magnesium, phosphorus, iron, copper, and zinc.

**Figure 1:**
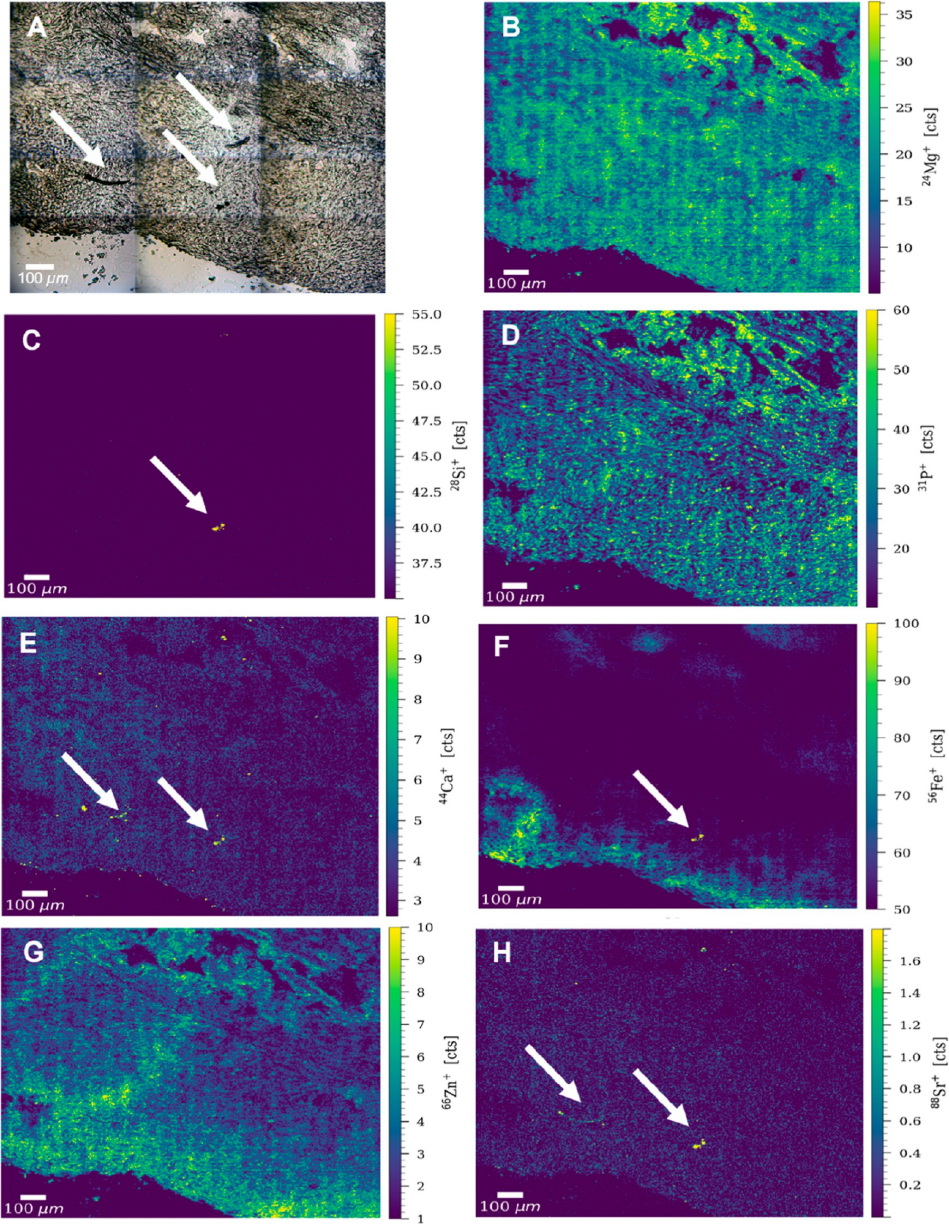
(A) Bright-field image of an ROI of the lung tissue sample from patient 1. Possible impurities are indicated by white arrows. Signal intensity maps of (B) ^24^Mg^+^, (C) ^28^Si^+^, (D) ^31^P^+^, (E) ^44^Ca^+^, (F) ^56^Fe^+^, (G) ^66^Zn^+^, and (H) ^88^Sr^+^, obtained by LA–ICP–TOFMS imaging. The fiber type structure and other impurities are indicated by white arrows.

Based on the bright-field images taken prior to LA, different ROIs with visible black impurities were selected for detailed and high-resolution LA–ICP–TOFMS analysis of human MPM lung tissue samples from two patients. The lung tissue sample of patient 1 showed distinct elemental distributions of magnesium, phosphorus, iron, and zinc, which allowed the visualization of the tissue structure (Figure 1). In one of the ROIs of the lung tissue sample, elevated levels of Si, Ca, Fe, and Sr well above the signals of the biological (tissue) background were found in distinct features (Figure 1). One of them resembled a long fiber (with a total length of around 200 μm) comparable to the asbestos fibers in the 2D cell samples, whereas the other features were round and relatively small (with a size of around 25–30 μm in diameter). The high spatial resolution of 2 μm allowed for the detection of small impurities, possible fiber fragments, or shorter mineral fibers. The long fiber could be visualized using the ^44^Ca^+^, ^56^Fe^+^, and ^88^Sr^+^ signals, whereas the other features were characterized by elevated Si, Ca, Fe, and Sr levels. Interestingly, one of the long impurities indicated by the white arrows in the bright-field image (Figure 1, panel A) yielded no significant counts of either of the metals, suggesting the presence of a non-asbestiform foreign body. This finding proves the capabilities of LA–ICP–TOFMS imaging to distinguish asbestiform fibers from other impurities always present in the human lungs. A noteworthy finding was the high amount of strontium yielded by two out of the three impurities present in the ROI and indicated by the white arrows (Figure 1, panel H). Sr has been previously reported as a potential toxic element (PTE) present in trace quantities in various types of asbestos, with a key role in MPM pathogenesis.^27^ One group has found high amounts of Sr, among other contaminants, in the hair samples of subjects environmentally exposed to crocidolite asbestos.^28^ However, quantitative analysis is further required to correctly classify the impurities in the investigated samples as crocidolite.

The fiber-type structure also displayed lower Mg and P levels than the surrounding tissue, whereas the Fe signal of the fibers/impurities proved to be around 20 times higher than the surrounding tissue signal (Figure 1, panels B, D, and F). To further confirm these results, the parts of the remaining long fiber and other fiber fragments, which were still visible on the glass slide after ablation with 1.0 J cm^–2^, were fully ablated using a significantly higher fluence of 3.0 J cm^–2^ (Figure S5). High levels of Ca, Fe, and Sr were detected by LA–ICP–TOFMS during the second ablation run. Mg signals generated by one of the impurities were noted following the second ablation run and the removal of the magnesium-enriched biological matrix (Figure S5). The increase in counts of some elements after ablation with a higher fluence (i.e., Fe and Mg) and the total lack of signal displayed for others (i.e., Si) highlighted the difficulty of spatially resolving mineral fibers (hard material) within a native biochemical environment (soft tissue). Furthermore, the spatial orientation of these fibers within the tumor microenvironment and translocation during sample preparation must also be considered.

To investigate the presence of more asbestos fibers and to get a more detailed picture of the metallome, a second ROI from patient 1 was ablated (Figure S6). Significantly elevated signals of magnesium, phosphorus, and zinc were noted on the proliferative leaf-like edges, indicating an increased metabolic activity.^29^ No mineral fibers (and no Si signals) were detected in this region, suggesting that fibers tend to accumulate in certain parts of the necrotic formation (Figure S6).

**Figure 2:**
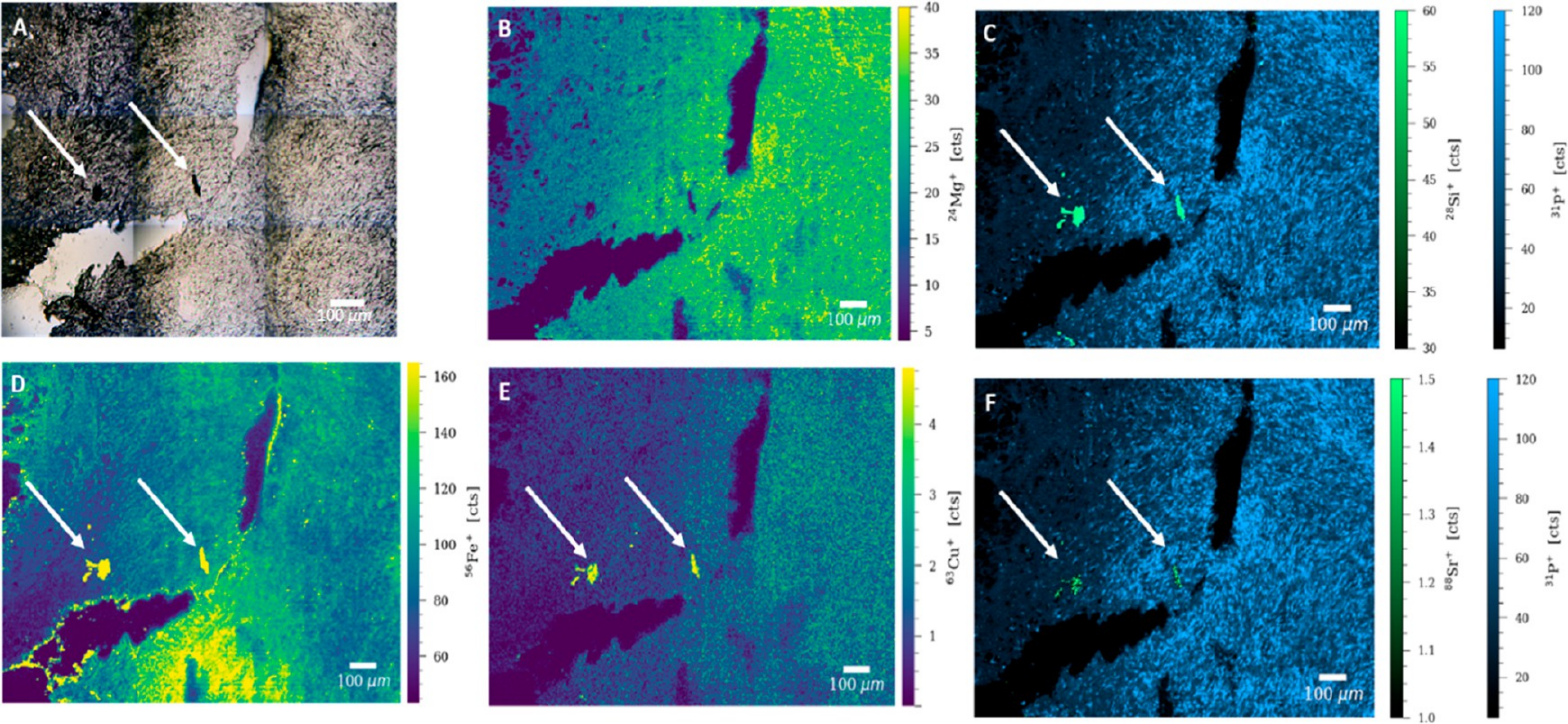
(A) Bright-field image of an ROI of the lung tissue sample from patient 2. Signal intensity maps of (B) ^24^Mg^+^, (C) an overlay of ^28^Si^+^ and ^31^P^+^, (D) ^56^Fe^+^, (E) ^63^Cu^+^, and (F) an overlay of ^88^Sr^+^ and ^31^P^+^, obtained by LA–ICP–TOFMS imaging. Possible impurities are indicated by white arrows.

In the lung tissue sample of patient 2, two clusters of black impurities were identified on the bright-field image, as indicated by the white arrows, and the ROI was subjected to LA–ICP–TOFMS analysis. The fibers had the typical appearance of ferruginous bodies and stained blue following Perls’ Prussian blue reaction, suggesting an increased presence of ferric iron coating the asbestos bodies (Figure S2). Asbestos bodies are not inert structures and are known to trigger endogenous metal mobilization across the tissue, most noteworthily of iron and copper.^13^ Iron sequestration by the malignant cells can be noted in Figure 2, panel D. Apart from the endogenous Cu known to coat asbestos fibers, Cu has been classified as an asbestos contaminant, also known as a PTE due to its abilities to increase the toxic character of asbestos fibers in a synergistic manner,^27,30^ which can be noted in Figure 2, panel E. Zinc levels also appeared to be elevated, with counts nearly twice as high as those of the rest of subjects (Figure S7). Zn is overabundant in the cancer tissue containing aggressive malignant cells in contrast to normal stroma.^31^ The high Si signals supported the hypothesis of the presence of asbestiform silicates. Similar to that for patient 1, the foreign bodies yielded no significant Na and Mg signals (Figures S7 and 2) following the first set of analyses. Moreover, high Sr levels were also recorded for these impurities, which strongly suggests the key roles of this PTE in MPM carcinogenesis (Figure 2, panel F). The sodium overabundance (over twice as high as that of the rest of the subjects) shown in Figure S7 can be linked to advanced MPM (stage IV).

### High-Resolution LA–ICP–TOFMS Imaging of the Pleura Tissue

**Figure 3:**
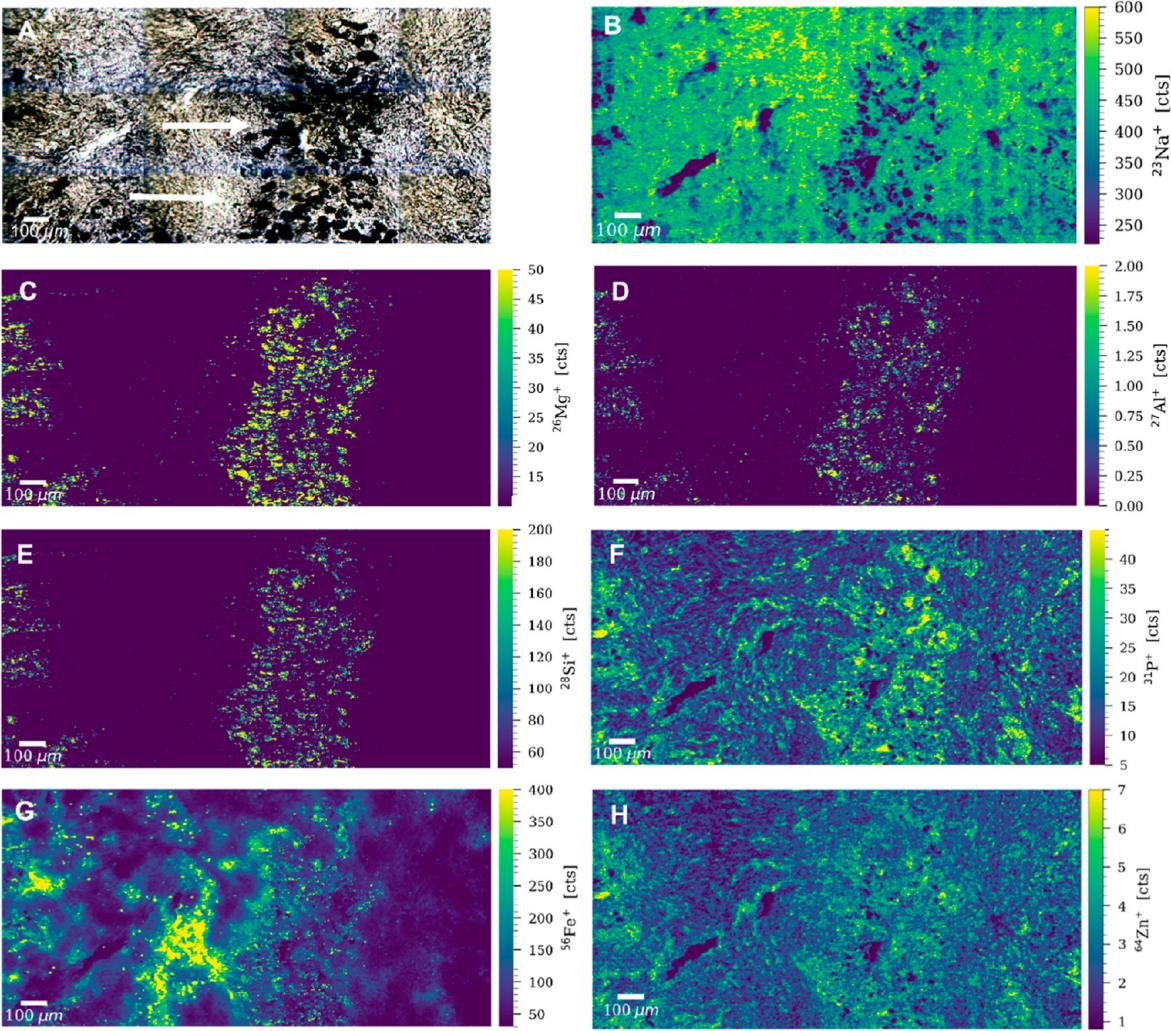
(A) Bright-field image of an ROI of the pleura sample of patient 3. Possible impurities are indicated by white arrows. Signal intensity maps of (B) ^23^Na^+^, (C) ^26^Mg^+^, (D) ^27^Al^+^, (E) ^28^Si^+^, (F) ^31^P^+^, (G) ^56^Fe^+^, and (H) ^64^Zn^+^, obtained by LA–ICP–TOFMS imaging.

Based on the bright-field images of the pleura sample from patient 3, ROIs were selected for detailed LA–ICP–TOFMS analysis. In this case, the black crystal-like structures (with a diameter of around 20–50 μm) that could be observed on the microscopic images of the pleura sample were of high interest for this study. Histological analysis revealed that these particulates did not stain for ferric iron (Perls’ Prussian blue staining) and did not retain color following H&E staining (Figures S1 and S2), which is characteristic to talc deposits stains.^32^ Additionally, the linear distribution and microscopic presentation of these particulates suggested the presence of talc deposits resulted from talc pleurodesis.^33^ In conjunction with LA–ICP–TOFMS imaging, the resulting data supported this hypothesis. Detailed LA–ICP–TOFMS analysis showed that these features were characterized by elevated magnesium and silicon levels and slightly increased aluminum and phosphorus levels (compared to that of the surrounding tissue, Figure 3, panels C–F). A further screening of the LA–ICP–TOFMS elemental distribution maps revealed that these structures also yielded some iron and zinc signals (Figure 3, panels G,H). In contrast to the fibers/impurities in the lung tissue, no distinct Sr signal was detected (data not shown). The general structure of talc can be expressed as Si_4_O_10_M_3_(OH)_2_, with Mg occupation at the M sites and replacement of Mg by ferrous iron (known as minnesotaite).^34^ The presence of ferrous iron explains why the particulates did not stain blue following Perls’ Prussian blue reaction, which reacts with ferric iron (Figure S1).

In terms of the pleura tissue metallome, sodium, phosphorus, and iron levels were among the most significant. First, elevated levels of Na can be visualized across the ROI (Figure 3, panel B). With essential roles in the osmolarity of the tumor microenvironment, an overabundance of sodium can impact the cell volume, metabolism, and immune processes.^35^ Moreover, high levels of Na can be associated with necrotic areas with poor vascularization, characteristic to the tumor microenvironment.^35^ Second, tumors are known to be phosphorus-demanding due to the increased ribosomal biogenesis in malignant cells, which requires a high amount of phosphorus.^36^ Clusters of high phosphorus signals of similar intensity to that of the talc particulates can be noted across the section (Figure 3, panel F). Interestingly, the distribution of the Fe signal appeared to be significantly increased around the talc deposits and in between clusters of these magnesium-rich particulates. Irritants such as talc can trigger an enduring state of inflammation,^32^ which in turn may dysregulate iron homeostasis in the lung.^37,38^ Iron sequestration by cancerous cells is also a hallmark of neoplastic transformation, which can explain the high Fe signals in the vicinity of the talc particulates (Figure 3, panel G).^39^

### High-Resolution LA–ICP–TOFMS Imaging of the Chest Wall

**Figure 4:**
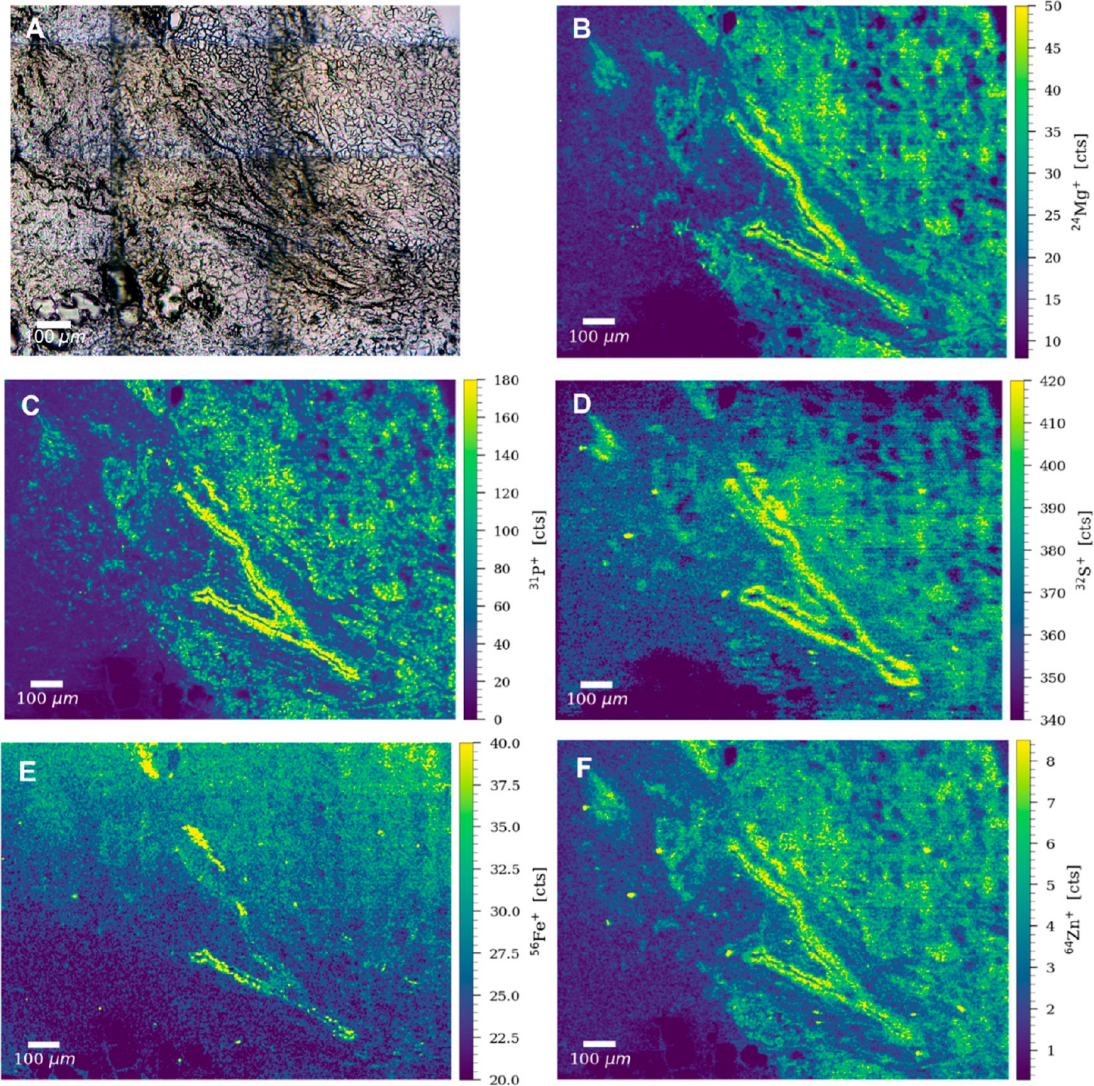
(A) Bright-field image of an ROI of the chest wall sample of patient 4. Signal intensity maps of (B) ^24^Mg^+^, (C) ^31^P^+^, (D) ^32^S^+^, (E) ^56^Fe^+^, and (F) ^64^Zn^+^, obtained by LA–ICP–TOFMS imaging.

Due to the increased pleural retention, asbestos fibers rarely tend to pass the parietal pleura and reach the chest wall.^40^ Accordingly, no asbestos fibers were detected in the chest wall section of patient 4. However, as patient 4 had a known history of edema and fibrosis (Table S2), the metallome of the chest wall was investigated by LA–ICP–TOFMS imaging. Selected ROIs included areas with possible fibrosis caused by prolonged chronic inflammation.^41^ Elevated signals of Mg, Fe, and Zn were recorded around the tissue scar (Figure 4). Magnesium and iron are known drivers of inflammatory responses by triggering cytokines, macrophages, and other immune cells,^39,42^ while zinc has been associated with increased cellular proliferation.^43^ Being an inflammation-driven mechanism characterized by cellular overgrowth, elevated levels of these metals were expected around the fibrotic regions (Figure 4, panels B, E, and F). Fibrosis is characterized by overgrowth, hardening, and/or scarring of the tissues and is attributed to excess deposition of the extracellular matrix.^41^ The elevated levels of P around the fibrotic tissue supported the “growth rate hypothesis”, which suggests that C/N/P ratios are influenced by the protein synthesis demand,^44^ and therefore, rapidly proliferating cells are rich in phosphorus (Figure 4, panel C). Interestingly, sulfur was also present across the tissue section, with significantly higher levels around the fibrotic tissue (Figure 4, panel D). The glycolytic metabolism is accelerated in cancer cells producing high levels of sulfur-rich compounds via the Maillard reaction.^45^

## Conclusions

Despite strict asbestos regulations being implemented in the Western World since the 1970s, MPM continues to be a burden to our societies.^46^ Asbestos detection is required to establish a causal link between the fibers and this malignancy; however, current asbestos identification techniques fail to preserve the spatial information and to detect fibers smaller than 5 μm.^47^ Additionally, there is little knowledge on the key functions that trace metals play in MPM progression.

Following a successful identification of asbestos and other mineral fibers within MPM cellular models by LA–ICPMS imaging, the analysis of human MPM tissue samples is a step further to integrating LA–ICPMS as a clinical tool. This study is the first attempt at mapping the multielement distribution of human MPM tissue samples with the aim of detecting asbestos and other mineral fibers, as well as getting insights into the tumor metallomics, with the use of LA–ICP–TOFMS imaging.

The initial step focused on optimizing the instrument parameters such as laser fluence and spot size by analyzing 2D MPM cellular models spiked with known types of asbestos fibers. Subsequently, tissue samples from four patients suffering from MPM of different pathologies and locations were analyzed using ultra-fast, high-resolution LA–ICP–TOFMS imaging. Asbestos fibers, most likely forming ferruginous bodies, were detected in two out of the four subjects, both of which were lung tissues. Interestingly, high levels of strontium, a PTE, were yielded by the impurities present in these samples following ablation. Strontium is not a commonly reported asbestos impurity, and there is limited research regarding its role in MPM onset and pathogenesis.^27,28^ Monitoring the metallome around the talc deposits offered insights into the impact talc pleurodesis has on tissues, and it is important to assess if the therapeutic benefits really outweigh the risks. The presence of talc particulates led to a significant increase in tissue damage and inflammation, as reflected by the high Fe signals, which were up to 10× higher than that for the rest of subjects. In terms of the metallome distribution, high levels of Mg, P, and Zn were associated with areas of high metabolic activity and increased proliferation, while Na overabundance was an indicative of poorly vascularized hypoxic regions.

Future work will focus on expanding the pool of subjects with the addition of benign and healthy tissue samples. Given the capabilities of the LA–ICP–TOFMS instrumentation, which allows for the monitoring of the entire mass range in a single run, biomarker discovery and validation can be attempted using metal-tagged antibodies.

## Supporting Information Available

Description of instrumental parameters for LA–ICP–TOFMS measurements, histology of tissue samples, bright-field images and LA–ICP–TOFMS elemental images of mesothelioma cells, LA–ICP–TOFMS elemental images of fibers in a lung tissue sample, and LA–ICP–TOFMS elemental images of an ROI of a lung tissue (PDF)
