# Supplementary material for: Elemental Mapping of Human Malignant Mesothelioma Tissue Samples Using High-Speed LA–ICP–TOFMS Imaging
Source: Anal Chem. 2022 Jan 24;94(5):2597–606. doi: 10.1021/acs.analchem.1c04857 (PMC8829826; doi:10.1021/acs.analchem.1c04857)
Supplement: Supplementary file 1 — ac1c04857_si_001.pdf [file ac1c04857_si_001.pdf]

# Supporting Information

## Elemental Mapping of Human Malignant Mesothelioma Tissue Samples using High-Speed LA-ICP-TOFMS Imaging

Oana M. Voloaca [a], Malcolm R. Clench [a], Gunda Koellensperger [b], Laura M. Cole [a], Sarah L. Haywood-Small [a], Sarah Theiner [b,*]

[a] Biomolecular Sciences Research Centre, Sheffield Hallam University, Howard Street, Sheffield, S1 1WB, United Kingdom

[b] Institute of Analytical Chemistry, Faculty of Chemistry, University of Vienna, Waehringer Straße 38, 1090 Vienna, Austria

Table of content

Table S1. Instrumental parameters for LA-ICP-TOFMS measurements.

Table S2. Patient details.

Figure S1 and Figure S2. Tissue histology following H&E staining and Perls' Prussian Blue staining.

Figure S3 and Figure S4. Bright-field images and LA-ICP-TOFMS elemental images of regions of interest of mesothelioma cells.

Figure S5. LA-ICP-TOFMS elemental images of fibers in a lung tissue sample.

Figure S6 and Figure S7. LA-ICP-TOFMS elemental images of a region of interest of a lung tissue.

**Table S1.** Instrumental parameters for LA-ICP-TOFMS measurements.

| | **LA-ICP-TOFMS** |
|---|---|
| RF Power [W] | 1450 |
| Sampling depth [mm] | 3.5 |
| Cone materials | Ni |
| Plasma gas flow [L $min^{-1}$] | 15.0 |
| Auxiliary gas flow [L $min^{-1}$] | 0.80 |
| Nebulizer gas flow [L $min^{-1}$] | 0.90 |
| m/z range | 14-256 |

| Patient no | Age | Gender | Tissue of Origin/Anatomic site | Sample pathology | TNM | Minimum stage grouping | % Normal | % Lesion | % Tumor | % Tumor Hypercellular Stroma | % Tumor Hypo/Acellular Stroma | Pathology Notes | Mineral fibres detected Yes/No |
|---|---|---|---|---|---|---|---|---|---|---|---|---|---|
| 1 | 62 | Male | Pleura / Lung | Tumor Mesothelioma of pleura, mixed | pT3pN1pMX | III | 0 | 0 | 90 | 5 | 5 | Pleural mesothelioma with extension along interlobular septa and nodular parenchymal deposits | Yes |
| 2 | 53 | Male | Pleura / Lung | Mesothelioma of pleura | pT3pN1pM1 | IV | 0 | 0 | 65 | 0 | 35 | Tumor Stroma (Hypo/Acellular): Fibrosis; Pleural mesothelioma with extension along interlobular septa | Yes |
| 3 | 65 | Male | Pleura / Pleura | Mesothelioma of pleura, epithelial | pT2pN2pMX | III | 0 | 0 | 25 | 75 | 0 | Tumor: epithelial variant; Tumor Stroma (Cellular): Fibroblastic cells, Inflammatory cells | Yes |
| 4 | 81 | Male | Pleura/Chest wall | Mesothelioma of pleura, epithelial. | Not reported | Not reported | 0 | 0 | 85 | 15 | 0 | Tumor Stroma (Hypo/Acellular): Fibrosis, edema | No |

**Table S2.** Patient details.

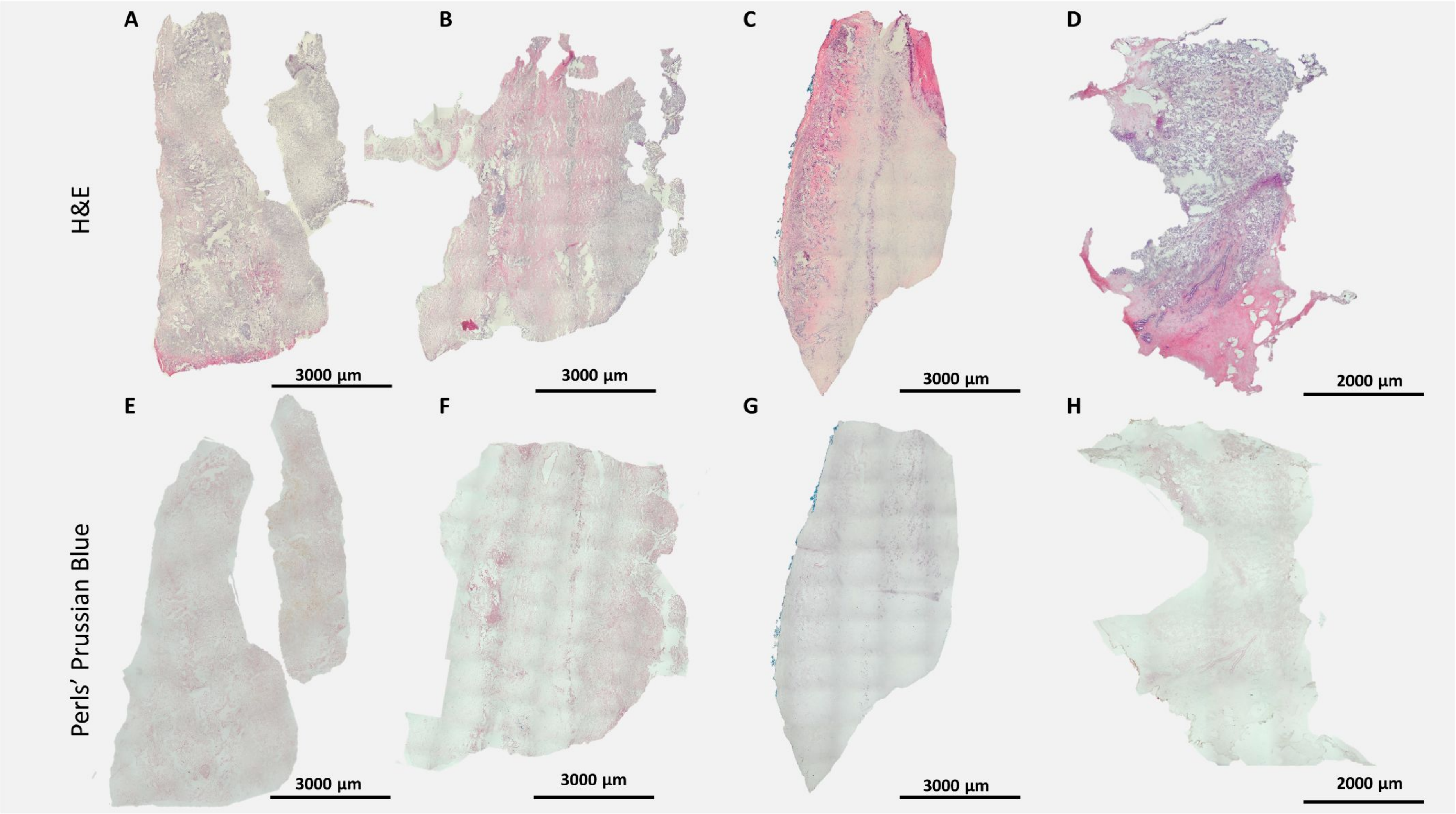
A
B
C
D
H&E
3000 µm
3000 µm
3000 µm
2000 µm
E
F
G
H
Perls' Prussian Blue
3000 µm
3000 µm
3000 µm
2000 µm

**Figure S1.** Tissue histology following H&E staining (A-D) and Perls' Prussian Blue staining (E-H). Patient 1&2- lung; Patient 3- pleura; Patient 4- chest wall. (A-D) Nuclei are stained purple by hematoxylin, whilst eosin stains cytoplasm and extracellular matrix pink. (E-H) Nuclei are stained pink with Nuclear Fast Red. The ferric iron present in asbestos reacts with potassium ferrocyanide and forms blue ferric ferrocyanide. The blue staining in panel (C&G) is caused by edge effect.

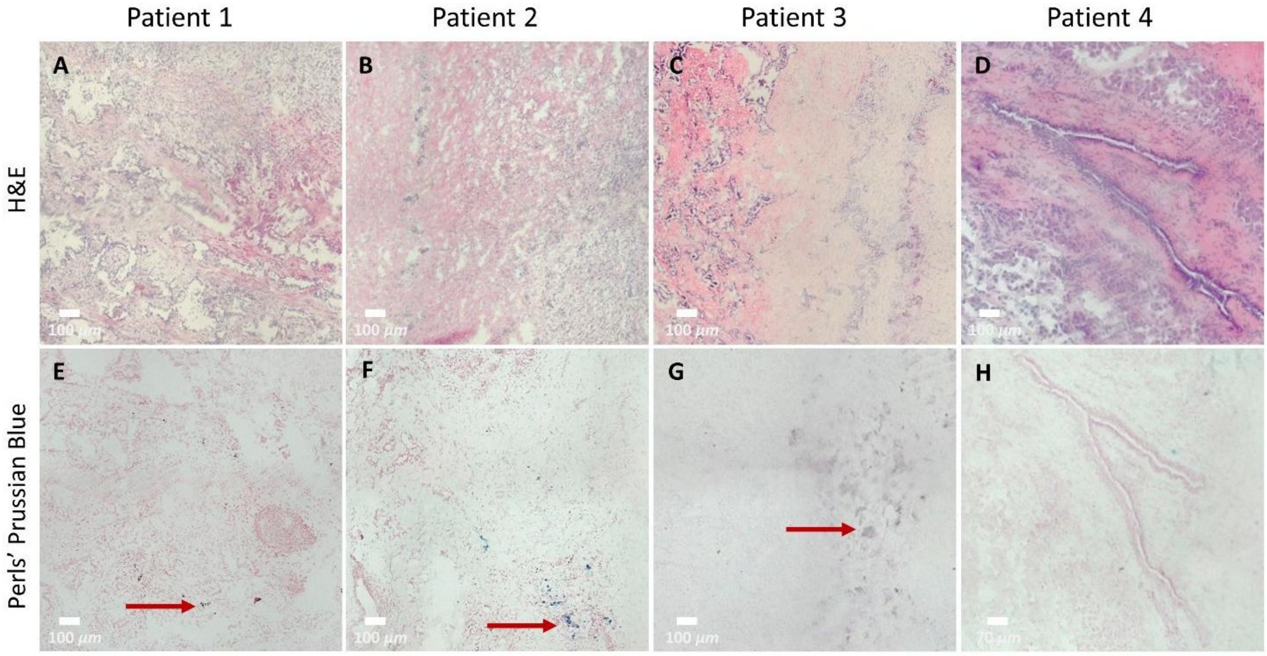


**Figure S2.** Tissue histology following H&E staining (A-D) and Perls' Prussian Blue staining (E-H). (A) Mixed mesothelioma, with spindle cells more prominent at the top region. (B) Desmoplastic growth present in the lower left corner, with areas of aggressive, spindle-like cells in the rest of the area. (C) Talc deposits appear to be less distinguishable following H&E staining. (D) Area of fibrosis is highlighted by the intense purple coloration. Traces of oedema can also be noted, mainly in the stroma. (E) Possible uncoated asbestos fibres are indicated by the arrow. (F) Coated ferruginous bodies are shown in blue. The size and distribution correspond to asbestos bodies deposition. (G) Red arrow points at the talc deposits that did not stain for ferric iron.

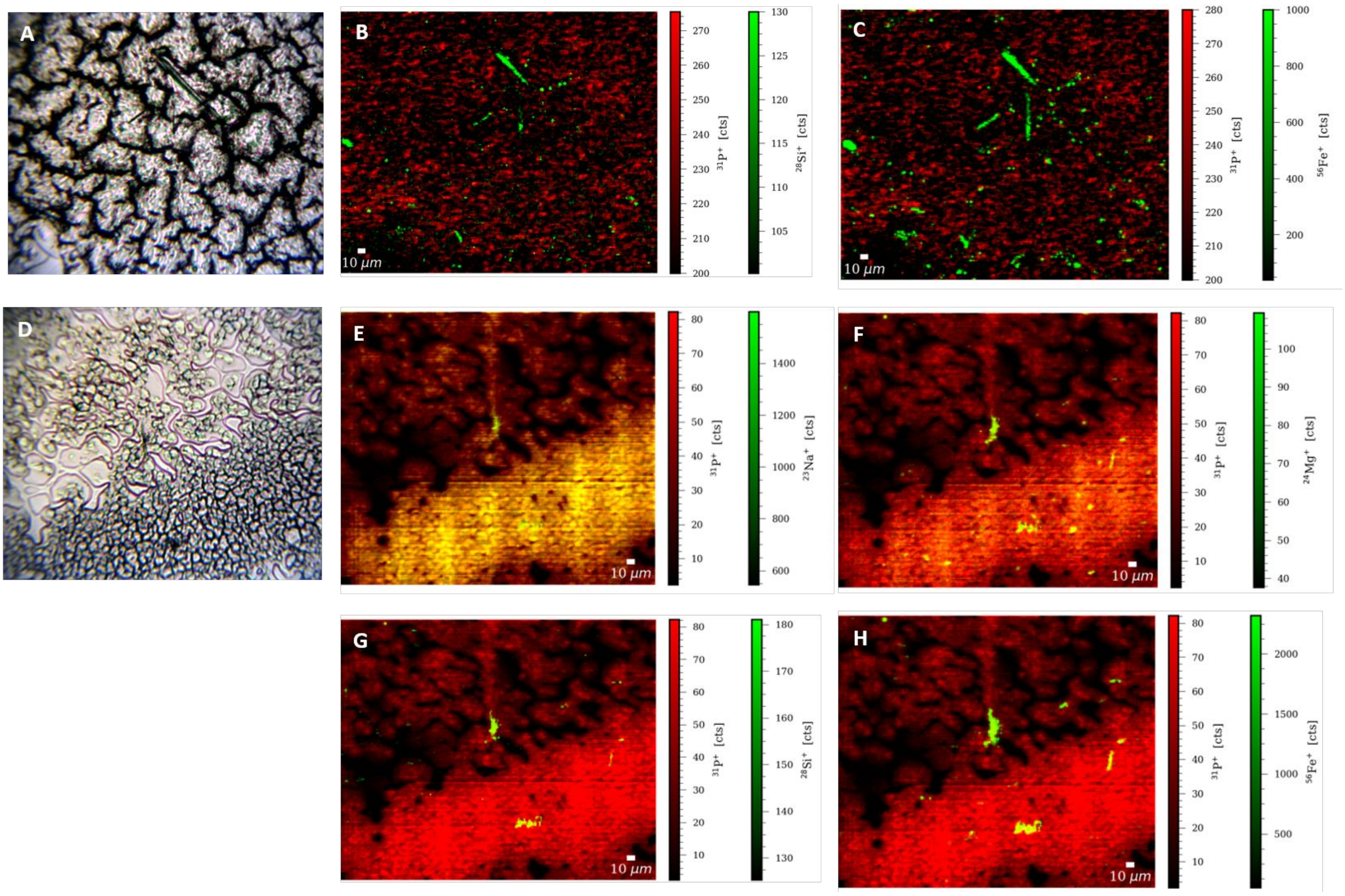


**Figure S3.** Bright-field images of regions of interest of mesothelioma cells spiked with (A) amosite and (D) crocidolite. Overlay of the signal intensity maps of $^{31}P^{+}$ with (B) $^{28}Si^{+}$, (C) $^{56}Fe^{+}$, (E) $^{23}Na^{+}$, (F) $^{24}Mg^{+}$, (G) $^{28}Si^{+}$, and (H) $^{56}Fe^{+}$, obtained by LA-ICP-TOFMS imaging.

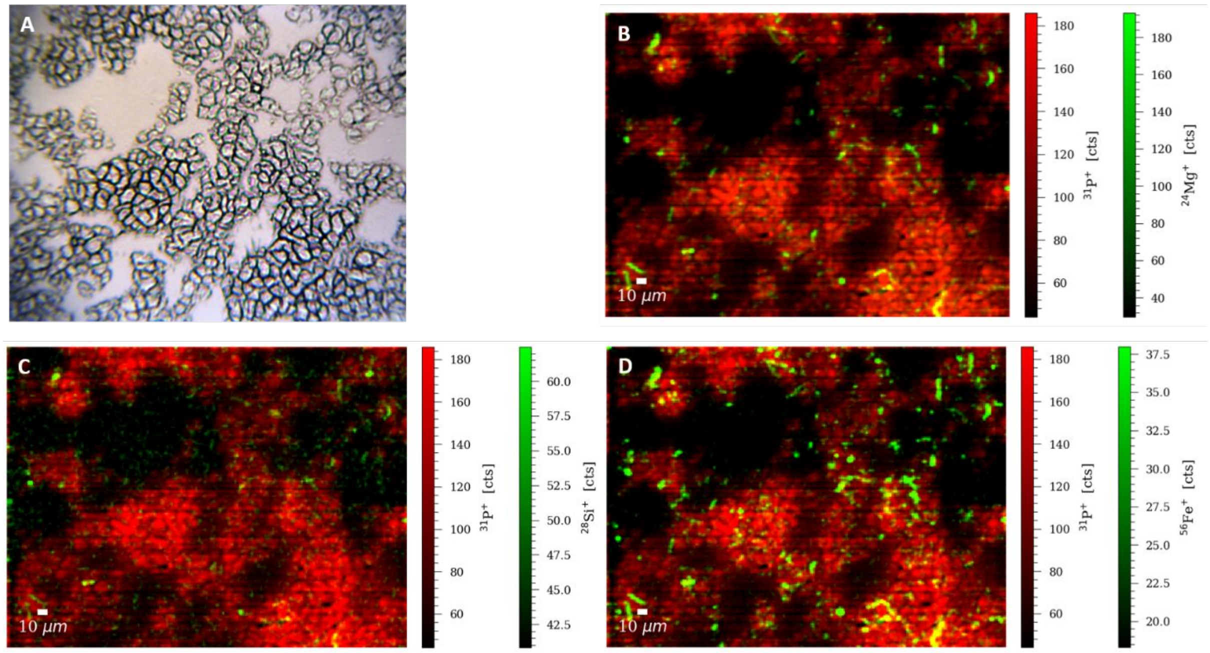


**Figure S4.** Bright-field image of regions of interest of mesothelioma cells spiked with (A) chrysotile. Overlay of the signal intensity maps of $^{31}P^{+}$ with (B) $^{24}Mg^{+}$, (C) $^{28}Si^{+}$, and (D) $^{56}Fe^{+}$, obtained by LA-ICP-TOFMS imaging.

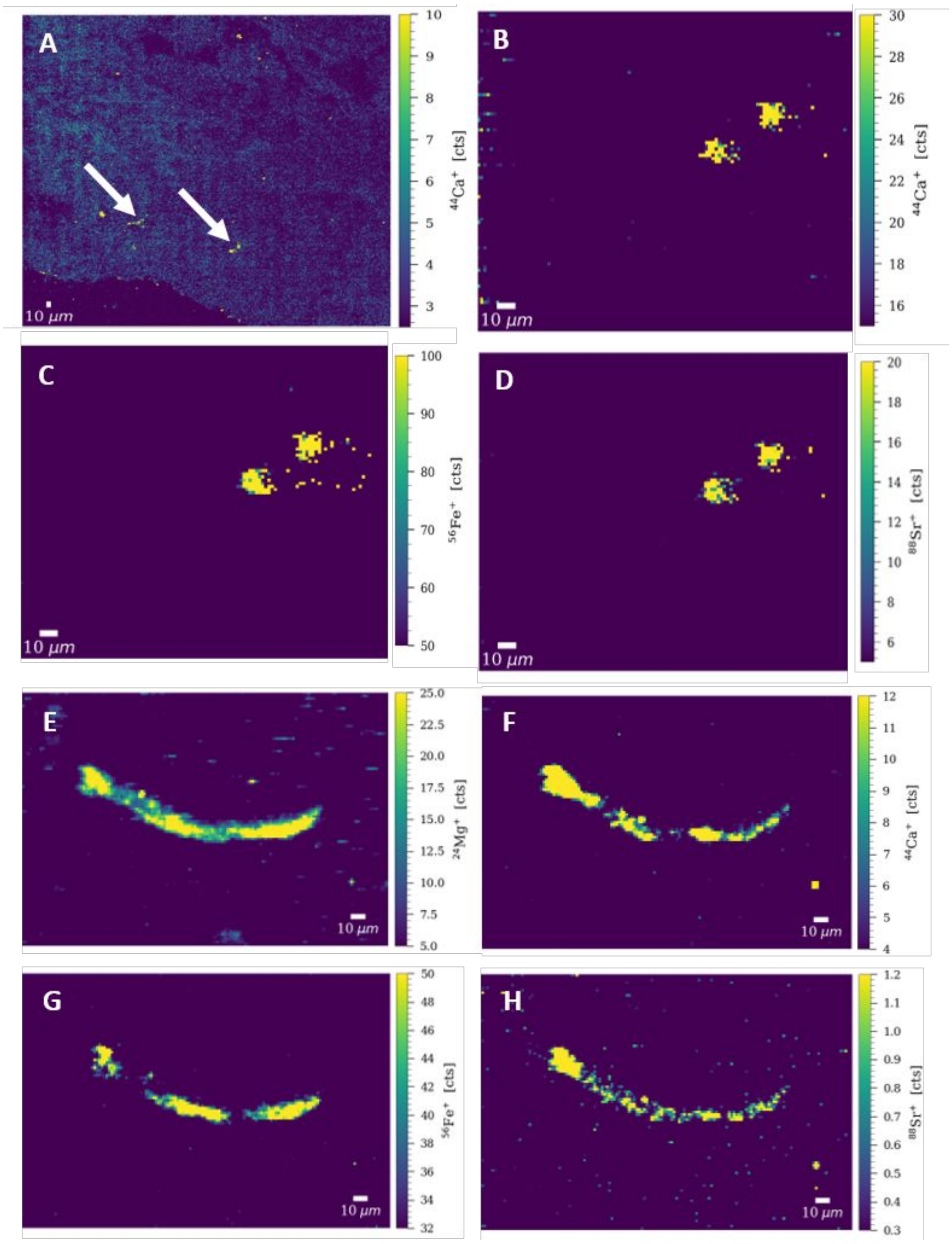


**Figure S5.** Signal intensity maps of (A) and (B) $^{44}Ca^+$, (C) $^{56}Fe^+$, (D) $^{88}Sr^+$, (E) $^{24}Mg^+$, (F) $^{44}Ca^+$, (G) $^{56}Fe^+$, and (H) $^{88}Sr^+$ of fibre fragments and a long fibre in the lung tissue sample of patient 1, obtained by LA-ICP-TOFMS imaging. The following laser ablation parameters were used: a repetition rate of 250 Hz, a pixel size of 2 μm and a fluence of 3.0 J $cm^{-2}$.

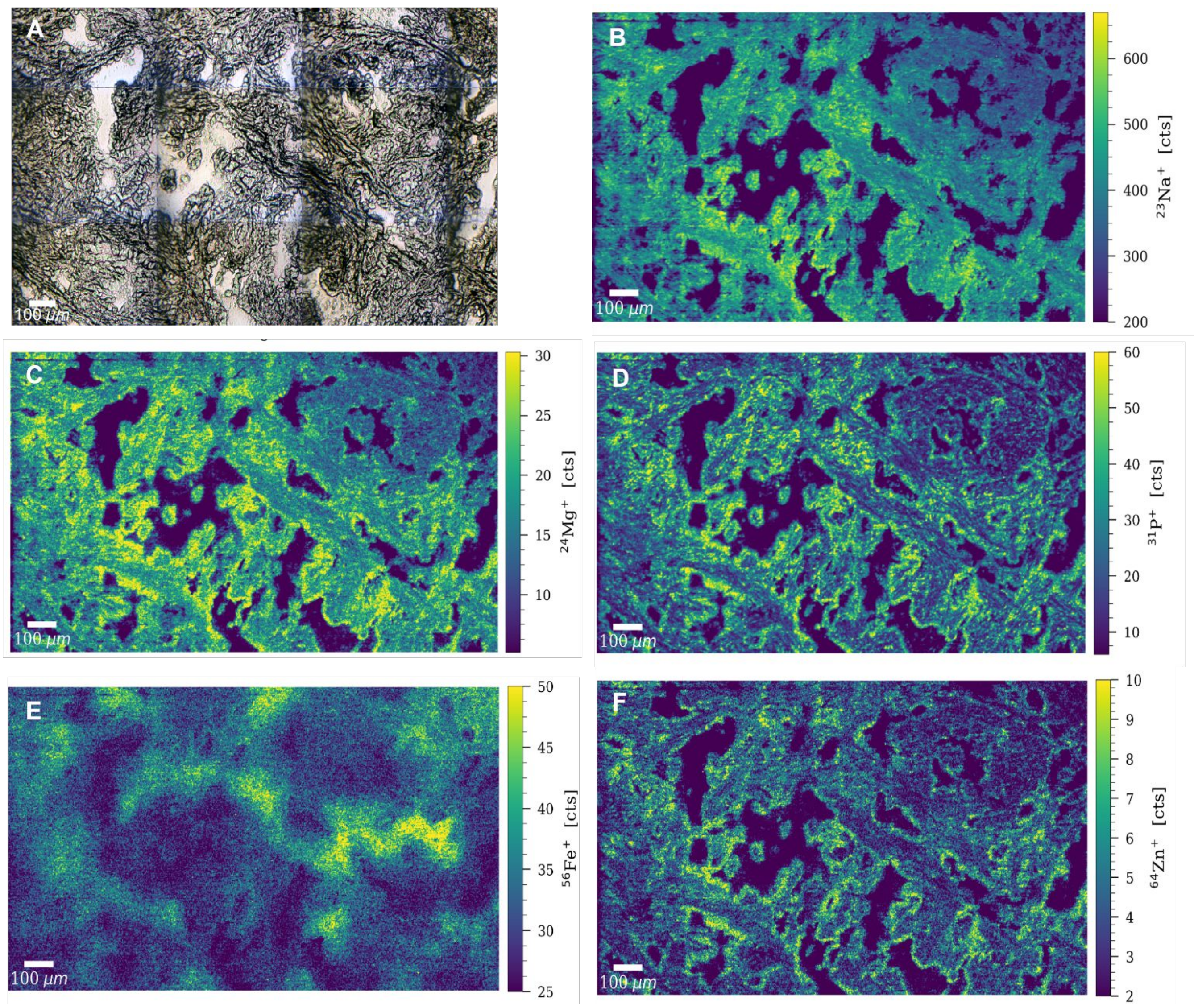


**Figure S6. (**A) Bright-field image of a region of interest of a lung tissue sample from patient 1. Signal intensity maps of (B) $^{23}Na^+$, (C) $^{24}Mg^+$, (D) $^{31}P^+$, (E) $^{56}Fe^+$, and (F) $^{64}Zn^+$, obtained by LA-ICP-TOFMS imaging.

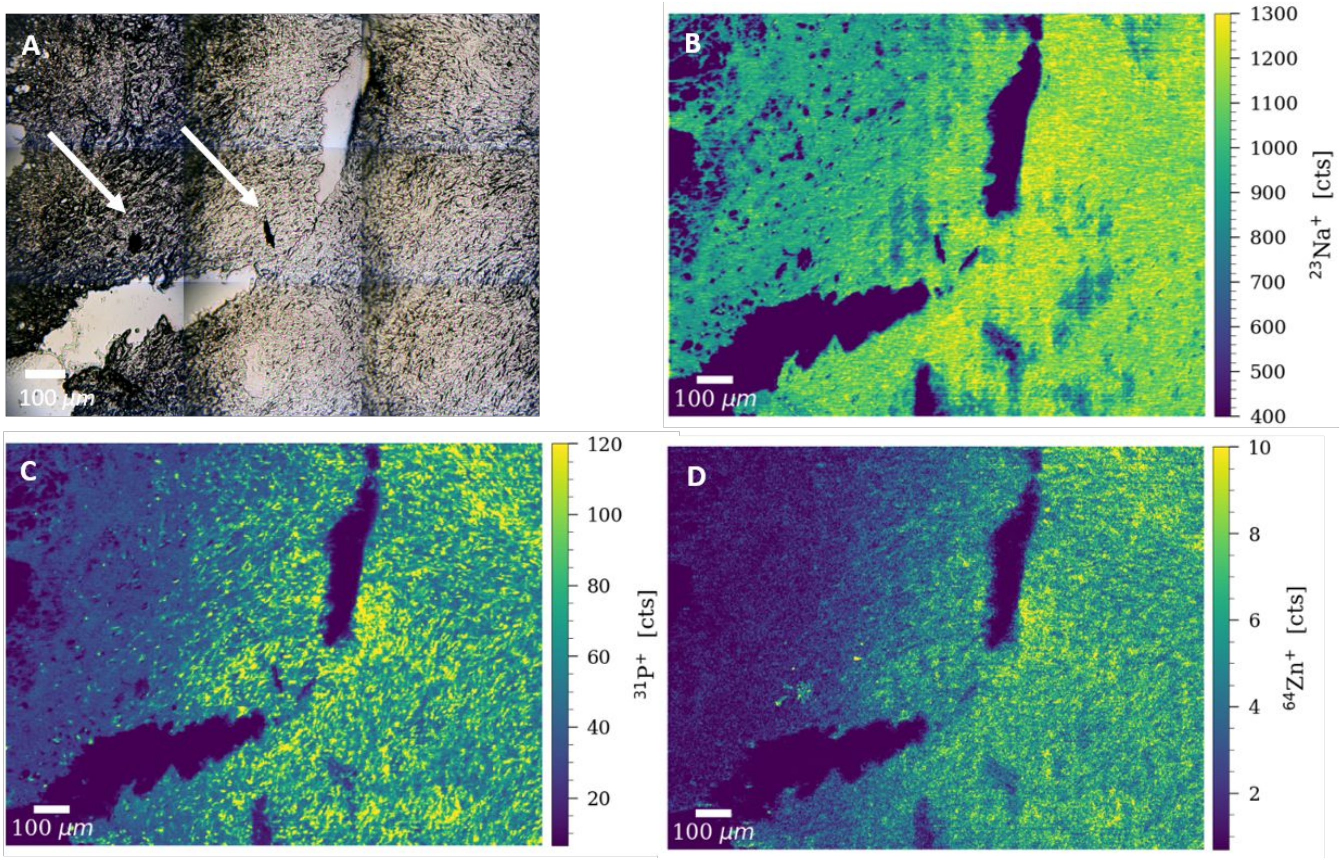


**Figure S7.** (A) Bright-field image of a region of interest of a lung tissue sample from patient 2. Signal intensity maps of (B) $^{23}Na^+$, (C) $^{31}P^+$, and (D) $^{64}Zn^+$, obtained by LA-ICP-TOFMS imaging.
